# Quantum Dots for Resistive Switching Memory and Artificial Synapse

**DOI:** 10.3390/nano14191575

**Published:** 2024-09-29

**Authors:** Gyeongpyo Kim, Seoyoung Park, Sungjun Kim

**Affiliations:** Division of Electronics and Electrical Engineering, Dongguk University, Seoul 04620, Republic of Korea

**Keywords:** quantum dot, resistive switching, switching mechanism, artificial synaptic device

## Abstract

Memristor devices for resistive-switching memory and artificial synapses have emerged as promising solutions for overcoming the technological challenges associated with the von Neumann bottleneck. Recently, due to their unique optoelectronic properties, solution processability, fast switching speeds, and low operating voltages, quantum dots (QDs) have drawn substantial research attention as candidate materials for memristors and artificial synapses. This review covers recent advancements in QD-based resistive random-access memory (RRAM) for resistive memory devices and artificial synapses. Following a brief introduction to QDs, the fundamental principles of the switching mechanism in RRAM are introduced. Then, the RRAM materials, synthesis techniques, and device performance are summarized for a relative comparison of RRAM materials. Finally, we introduce QD-based RRAM and discuss the challenges associated with its implementation in memristors and artificial synapses.

## 1. Introduction

**Figure 1:**
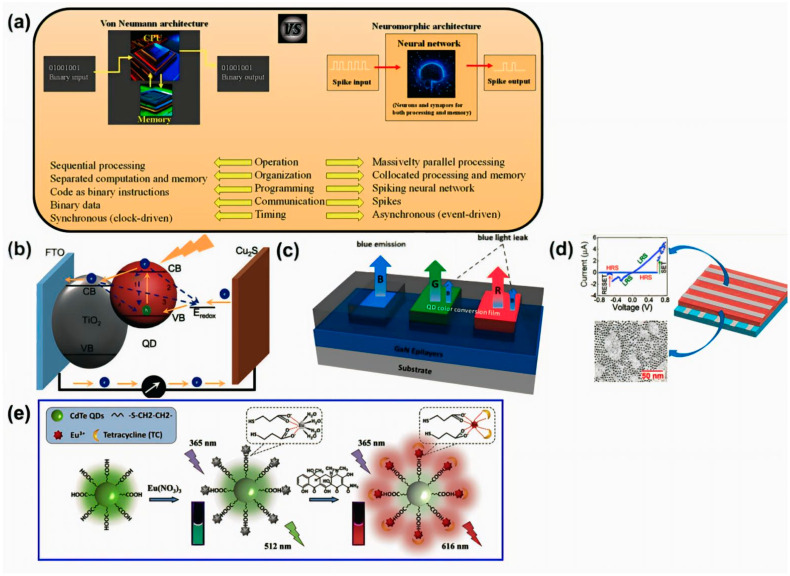
(**a**) Comparison between von Neumann architecture and neuromorphic architecture [25]. Copyright 2023, MDPI. (**b**) Schematic illustration of carrier dynamics (generation, separation/transport, and recombination in QDs) [26]. Copyright 2020. Wiley Online Library. (**c**) Schematic illustration of QD color conversion layer on GaN-based blue micro-LEDs [27]. Copyright 2021, ACS Publications. (**d**) Illustration of Al/CdSe-PVP/Al resistive memory device along with TEM image of synthesized QDs and I–V curve [28]. Copyright 2022, ACS Publications. (**e**) Preparation of Eu/CdTe QD nanostructure and sensing mechanism for tetracycline [29]. Copyright 2020, Elsevier.

With the Fourth Industrial Revolution and the rapid advancement of AI technology, data production has reached levels that are challenging to handle with traditional von Neumann systems; thus, maintaining Moore’s Law has become difficult. To address this issue, neuromorphic technology, and artificial synapses, which mimic the human brain, have been proposed as solutions [1,2]. The concept of building electronic systems that replicate the functions of the biological nervous system is currently attracting significant interest. The advantages of the neuromorphic structure compared with the classical von Neumann structure are shown in Figure 1a. However, as the human cerebral cortex contains approximately 10^14^ synapses, implementing hardware that uses large-scale parallel and compact electronic systems is extremely challenging [3]. In this regard, resistive random-access memory (RRAM) represents a potentially viable next-generation memory technology for implementing artificial synapses, due to its fast switching speed, low power consumption, nonvolatile characteristics, and high scalability [4,5]. RRAMs typically have a metal–insulator–metal (MIM) structure, with metal oxides commonly used as the insulator layer. RRAM with organic materials and 2D materials has been widely studied [6,7], with quantum dot (QD)-based RRAM gaining particular attention due to its various advantages.

Among the materials used for RRAM, nanometer-sized semiconductor particles called QDs are promising for various applications, such as solar cells, light-emitting diodes (LEDs), sensors, and memory, due to their excellent optical properties and solution processability [8,9], which are attributed to their ability to emit or absorb specific wavelengths [10,11]. Solution-processable inorganic semiconductor QDs maintain the excellent electronic performance and structural stability of crystalline inorganic materials while being processed in solution; hence, they represent a reasonable choice for fabricating nonvolatile memories [12,13]. In particular, QD-based RRAM combines the advantages of RRAM—simple structure, easy operation, low power consumption, and nonvolatility—with the nanoscale and miniaturization opportunities offered by QDs, thereby attracting significant attention from both academia and industry [14,15]. Additionally, the performance of QD-based RRAM can be further enhanced by using uniform oxides [15]. Furthermore, QD-based RRAM can be implemented as optically tunable memory [16], which highlights the potential of this technology. Inorganic semiconductor quantum dots (QDs) present several significant disadvantages that impede widespread adoption and commercialization. A primary concern is the presence of toxic elements such as lead (Pb) and cadmium (Cd) in many inorganic QDs, which pose substantial risks to both environmental safety and human health. Furthermore, the stability of inorganic QDs is often compromised over time, particularly due to factors such as oxidation or exposure to heat. For instance, air or moisture can significantly reduce the stability of inorganic semiconductor quantum dots, leading to performance degradation. Additionally, certain inorganic semiconductor QDs exhibit limited optical properties, performing optimally only within specific wavelength ranges, which constrains applicability across broader applications. Such limitations collectively hinder the extensive use and commercial viability of inorganic semiconductor QDs. Nevertheless, ongoing research and development efforts are being directed toward mitigating these challenges. Researchers are exploring the development of non-toxic QDs as alternatives to hazardous compounds, aiming to enhance the safety profile of these materials. In parallel, advancements in synthesis techniques and production processes are being pursued to improve the stability of inorganic QDs. Technological innovations are also focusing on precise control over the size and shape of QDs, as well as the investigation of novel materials and structures that could broaden the applicability of inorganic semiconductor quantum dots. Should the research endeavors prove successful, inorganic semiconductor QDs may find expanded applications and play a pivotal role in the development of next-generation electronic and optoelectronic devices. Recent research has shown that artificial synapses can be activated through light stimulation, which elucidates and validates the concept of optogenetically designed neurons [17,18]. Considering the excellent optical properties of QDs, QD-based electronic materials could be advantageous in fabricating RRAM for artificial synapse implementation.

Research is being actively conducted on artificial synapse implementation using QD-based RRAM [19,20]. Simulations have demonstrated the capability of QD-based RRAM to mimic biological synapses; examples include paired-pulse facilitation (PPF) [21]—a well-known form of short-term synaptic plasticity exhibited by biological synapses—which plays a crucial role in temporal information processing, and the spike-timing-dependent plasticity (STDP) learning rule [22,23], which regulates the connection strength between neurons in the brain [24].

This review summarizes the recent advancements in QD-based RRAM. It discusses the key material properties and fabrication methods for QDs and introduces the major RRAM mechanisms from various perspectives, including Schottky emission, the space-charge-limited current mechanism, and the Poole–Frenkel mechanism. Next, the advantages of RRAM and the types of materials used to construct RRAM are explored, with a focus on QDs. The recent research trends in QD-based RRAM are then examined and evaluated. Finally, instances of QD-based RRAM being used as artificial synapses are discussed, their potential is assessed, and their application prospects are identified. This review aims to provide guidance and inspiration for developing more advanced QD-based RRAMs.

## 2. Quantum Dots

QDs exhibit quantum confinement effects and thus display unique material properties, including photoinduced electron transfer, electron storage capabilities, and upconversion characteristics; thus, QDs are highly versatile [30]. These distinctive properties arise from their capability to emit or absorb light at specific wavelengths [31,32]. Additionally, QDs are noted for being highly photosensitive and multifunctional, even at very small feature sizes, while maintaining structural stability and being processable in solution [33]. Hence, integrating QDs with other material systems can address the inherent limitations of devices based on single materials [34,35]. For example, as shown in Figure 1b–e, QDs have been used in flash memory, solar cells, energy storage, sensors, and RRAM and are deemed promising in various fields. Upon examining Figure 1b, QDs offer numerous advantages in the solar cell industry. First, the ability to absorb light at various wavelengths depending on size allows for efficient utilization of a broad spectrum of sunlight. Additionally, QDs exhibit high electron mobility, which enhances conversion efficiency in transforming absorbed light into electrical energy. The nanoscale fabrication of QDs permits a wide range of shapes and configurations, enabling potential applications on flexible substrates. Moreover, QDs can be synthesized from relatively inexpensive raw materials, facilitating large-scale production. The use of core/shell architectures reduces surface defects, thereby improving stability and extending the lifespan of solar cells. Such advantages position QDs as key components in the development of next-generation solar cell technologies. As shown in Figure 1c, The quantum dot (QD) color conversion layer presents significant energy storage benefits for micro-LED displays. QDs possess unique optical properties that enable simultaneous light absorption and color conversion. The study demonstrates that QD films can achieve color conversion efficiencies of 25% for red and 4.5% for green, while suppressing residual blue light by 0.1%, even at thicknesses below 20 μm. Such properties are enhanced at high QD concentrations and low film thicknesses, effectively storing and converting energy to increase emission intensity. Notably, the addition of TiO_2_ nanoparticles to the QD matrix resulted in more than a 10% improvement in emission intensity. The use of a QD-based color conversion layer optimizes color conversion efficiency while providing energy storage and utilization benefits, contributing to the production of cost-effective displays. Such properties are enhanced at high QD concentrations and low film thicknesses, effectively storing and converting energy to increase emission intensity. To describe Figure 1d, Resistive random access memory (RRAM) leverages quantum dots (QDs) as low-power, high-performance memory components. The non-volatile resistive memory characteristics of metal chalcogenide-based CdSe QDs can be finely controlled through surface passivation with different ligands and tuning of electronic states. Recent studies have demonstrated that Al/CdSe-PVP/Al RRAM devices can be fabricated by combining the organic polymer poly(4-vinylpyridine) (PVP) with high-quality CdSe QDs synthesized via a high-temperature injection method. Such devices exhibit a high current ON/OFF ratio of 6.1 × 10^4^, fast switching speeds, and extended retention times. Furthermore, excellent bipolar non-volatile resistive switching performance is demonstrated at very low switching voltages (<±0.8 V). Such characteristics make CdSe-PVP-based RRAM devices promising candidates for high-performance, low-power memristors. As illustrated in Figure 1e, Recently, significant interest has emerged in developing sensors using metal ions and quantum dots (QDs). In the study, europium ions (Eu^3+^) were used as functionalized ligands, and cadmium telluride quantum dots (CdTe QDs) served as the matrix material to fabricate a dual-responsive fluorescence sensor for detecting tetracycline (TC). The sensor operates in two stages. In the first stage, Eu^3+^ binds to the CdTe QD surface, leading to the formation of a hybrid nanosensor. Such interaction causes a decrease in the green fluorescence emitted by the CdTe QDs. In the second stage, when TC is introduced, the compound binds to Eu^3+^ and facilitates energy transfer, resulting in red fluorescence emission from Eu^3+^. As the TC concentration increases, the fluorescence of CdTe QDs continues to decrease while Eu^3+^ red fluorescence intensifies, causing the sensor to change from green to bright red. The study highlights the potential of CdTe QD and Eu^3+^-based sensors for real-time detection of contaminants in food safety and environmental protection applications.

### 2.1. Material Properties of QDs

**Figure 2:**
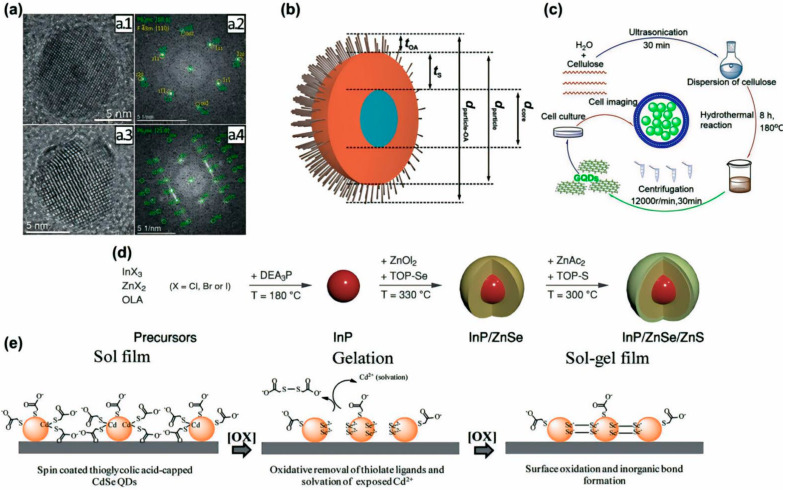
(**a**) Results of analysis of core–shell structure with high-resolution transmission electron microscopy (HR-TEM). Determination of crystal structure based on HR-TEM images of individual CdSe/CdS particles, shown in Panels (**a.1**) and (**a.3**), and the resulting Fourier transformations, shown in Panels (**a.2**) and (**a.4**), respectively, superimposed on the corresponding simulated diffraction patterns. The jems software program was used to simulate the diffraction patterns. (**b**) Model of CdSe/CdS QDs (tOA: thickness of oleylamine ligand shell; tS: thickness of CdS shell; dcore: diameter of CdSe core; dparticle: diameter of particle that is unequivocally detectable with TEM and SAXS; dparticle-OA: diameter of particle, including organic shell) [42]. Copyright 2020, nature.com. (**c**) Sustainable route for green synthesis of GQD from cellulose [43]. Copyright 2019, Wiley Online Library. (**d**) Synthesis scheme for InP/ZnSe/ZnS QDs using indium and zinc halides, tris(diethylamino)phosphine, zinc carboxylates, tri-n-octylphosphine selenide, and sulfide in oleylamine and 1-octadecene [44]. Copyright 2022, ACS Publications. (**e**) Mechanism of oxidative (OX) gelation of thioglycolic acid-capped QD film (sol film) submerged in TNM solution [45]. Copyright, 2013.

QDs exhibit unique material properties due to the quantum confinement effects arising from their small size [36]. QDs can be captured in transmission electron microscope images, and their structure generally consists of a core and shell, as shown in Figure 2a,b. The properties and applications of quantum dots (QDs) are significantly influenced by the core-shell structure. The size and composition of the core directly affect the photoluminescence quantum yield (PLQY) and exhibit distinctive electrical and optical properties due to the combination of semiconductor materials. The shell, acting as a passivation layer around the core, greatly impacts the stability and optical performance of QDs. A sufficiently thick shell can increase PLQY and suppress non-radiative recombination. The characteristics are best exemplified by the CdSe/CdS core-shell configuration, where the shell thickness can be adjusted to optimize QD performance. Additionally, the shell minimizes the effects of external environments and passivates the core’s surface, enhancing colloidal stability and reliability in various applications, such as bioimaging and display technologies. Advanced analytical techniques, including X-ray photoelectron spectroscopy (XPS), dynamic light scattering (DLS), and high-resolution transmission electron microscopy (HR-TEM), are employed to measure the size, shape, distribution, shell thickness, and chemical composition of core and core-shell nanostructures. In summary, the properties and applications of QDs are closely tied to the core-shell structures, and optimizing QD optical performance begins with controlling shell thickness and chemical composition. The electronic properties of QDs change with size; smaller QDs possess larger bandgaps, which allow them to absorb and emit higher-energy light [37]. Thus, when stimulated, QDs emit light of specific wavelengths, and the color of the emitted light depends on the size of the QD. This characteristic is utilized in applications such as bioimaging and display technologies [38]. Additionally, because of their broad absorption spectrum, QDs can be employed in devices such as solar cells and photodetectors [39,40]. Furthermore, as QDs are generally more resistant to chemical decomposition than bulk materials or organic dyes, they are suitable for various industrial applications [36]. Depending on their composition and the embedding matrix, QDs can also exhibit significant mechanical flexibility and are, therefore, useful in flexible devices [41].

### 2.2. QD Manufacturing

#### 2.2.1. Recent Quantum Dot Synthesis Techniques

In recent years, various techniques have been employed for the synthesis of quantum dots (QDs), as summarized in Table 1. To enhance clarity, the techniques can be categorized into distinct groups. Solution-processable methods, such as sol-gel and colloidal synthesis, are commonly utilized for the fabrication of inorganic semiconductors. However, certain precursors and solvents used in colloidal synthesis present significant environmental and health hazards [46]. Although the sol-gel process can be conducted at low temperatures, the method suffers from drawbacks, including poor product homogeneity and extended reaction times [47]. On the other hand, techniques like thermal decomposition, chemical precipitation, and self-assembly are more suitable for solid-state processing or other specific categories. Thermal decomposition, for example, requires high reaction temperatures, leading to substantial energy consumption [48,49]. Chemical precipitation may involve the use of toxic substances, and complex methods such as self-assembly often necessitate highly controlled conditions. Most of the existing QD synthesis techniques are characterized by long reaction times, the use of hazardous chemicals, specialized equipment, or unfavorable reaction conditions. Consequently, current research efforts are increasingly focused on the development of more efficient, cost-effective, and environmentally sustainable synthesis methods. Notable examples of innovative approaches include green synthesis [50], sonochemical synthesis [51], electrochemical synthesis [52], and plasma synthesis [53]. Figure 2c–e provide a concise overview of the sol-gel, halide, and green synthesis processes.

#### 2.2.2. Synthesis Methods for QDs

**Figure 3:**
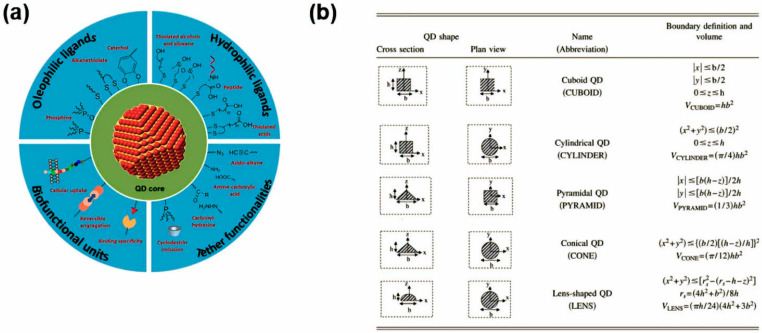
(**a**) Surface-engineering strategies for QDs for end-applications: capping layers with oleophilic ligands, water-soluble surface layers with hydrophilic ligands, versatile tether functionalities via selective reactions or interactions, and bio-functionalities for targeting or therapeutic applications [75]. Copyright 2017, Elsevier. (**b**) Quantum dot shapes considered [76]. Copyright 2006, American Physical Society.

Because QDs have a substantial surface area-to-volume ratio, their surface chemical bonding with ligands is crucial for their uniform dispersion during device fabrication. This uniform dispersion is vital as it affects the final performance of the devices. To prevent the agglomeration of QDs, which can be caused by large charged surfaces, coating the QD surface with polymer ligands is an effective strategy [69]. This coating facilitates the processing of thin-film memory devices through techniques such as spin-coating and printing. The performance of QD-based devices is significantly influenced by the charge-carrier transport within the QDs, especially interparticle and interface transport [70,71]. These aspects are critical to the electrical and optoelectronic properties of the device. Surface ligands on QDs are particularly important for the efficient charge transfer of photogenerated carriers. Therefore, extensive research has been conducted on various ligands to determine the best options for enhancing photosensitivity and photoconductive gain [72,73]. Figure 3a introduces various surface ligand powers. To select the optimal ligand, the strengths and weaknesses of different ligands must be evaluated in terms of strengthening the electronic bonding between neighboring QDs. Modifying parameters such as the ligand’s binding strength, chain length, polarity, and molecular structure can significantly improve the performance of QD-based devices [74]. To gain a deeper understanding of the synthesis methods for QDs, we will discuss the molecular beam epitaxy (MBE) growth techniques, specifically focusing on the Stranski–Krastanov (SK) growth mode and van der Waals epitaxy. The SK growth model, which is intimately linked to the development of van der Waals epitaxy (VdWE) and MBE, is a widely used approach for the synthesis of QDs. The growth mechanism of the SK model can be delineated into five distinct stages, each corresponding to the characteristics of both MBE and VdWE. The initial stage involves two-dimensional (2D) growth, where planar atoms adhere to the substrate surface. In MBE, the deposited atoms form a stable 2D structure through the formation of strong chemical bonds with the substrate. In contrast, in VdWE, the atoms bond weakly to the substrate via van der Waals interactions, leading to the formation of a 2D layer. As the 2D layer reaches a critical thickness, lattice constant discrepancies between the substrate and the deposited layer give rise to stress, marking the onset of the stress accumulation and instability development stage. When this stress exceeds a critical threshold, the growth mode transitions from 2D to three-dimensional (3D) island growth. In MBE, lattice mismatches facilitate the transition by promoting the accumulation of stress. In VdWE, the weak bonding to the substrate helps maintain lower stress levels, contributing to enhanced stability even with significant lattice mismatch. The third stage, 3D island growth, is characterized by the formation of atom clusters into three-dimensional islands. In MBE, individual islands continue to grow independently as atoms rearrange themselves on the island surfaces, minimizing energy. In VdWE, the weak attachment of the islands to the substrate allows for structural flexibility, enabling the formation and growth of islands in a low-defect, low-stress state. The stage leads to the development of islands that independently increase in size and attain more defined shapes. In the subsequent stage, the islands achieve a stable thickness and size, marking the equilibrium state. During the phase, the growth of QDs stabilizes, and the electrical and optical properties of QDs are optimized. The interaction between the crystal structure and the substrate plays a crucial role in defining the stage in both MBE and VdWE. By leveraging the advantages of both MBE and VdWE, the SK model facilitates the efficient synthesis of quantum dots, even in materials with significant lattice mismatch. The versatility enables the production of high-performance QDs suitable for a wide range of optoelectronic applications, including photodetectors, lasers, and solar cells.

#### 2.2.3. Size

QDs are typically 2–10 nm in size. In the study, a theoretical analysis of the electronic states of QDs with various sizes and shapes was conducted. Specifically, QDs were modeled using different geometries, such as cubic, cylindrical, pyramidal, conical, and lens-shaped structures, to examine the influence of size and shape on electronic states. Key findings indicate that the energy of electronic states decreases with increasing QD volume, which is attributed to a reduction in quantum confinement. Additionally, among QDs with the same volume, QDs with narrower ends exhibited higher ground state energies compared to QDs with wider ends, likely due to the smaller effective volume of the former. Researchers calculated the ground state energies for various QD shapes and validated results by comparing them with established theoretical and experimental data. Notably, it was predicted that lens-shaped QDs with smaller area ratios would transition into higher energy levels compared to lens-shaped QDs with larger area ratios. Regarding the significance of discrete energy levels, it was emphasized that QDs exhibit discrete energy levels due to the confinement of electrons and holes in three dimensions. The energy level spacing increases as the QD size decreases, and the QD shape further influences these energy levels. Findings suggest that by varying the size and shape of QDs, distinct electrical and optical properties can be achieved, providing crucial insights for the development of QD-based devices, such as lasers, infrared photodetectors, and electro-absorption modulators [76].

## 3. Resistive Random Access Memory

**Figure 4:**
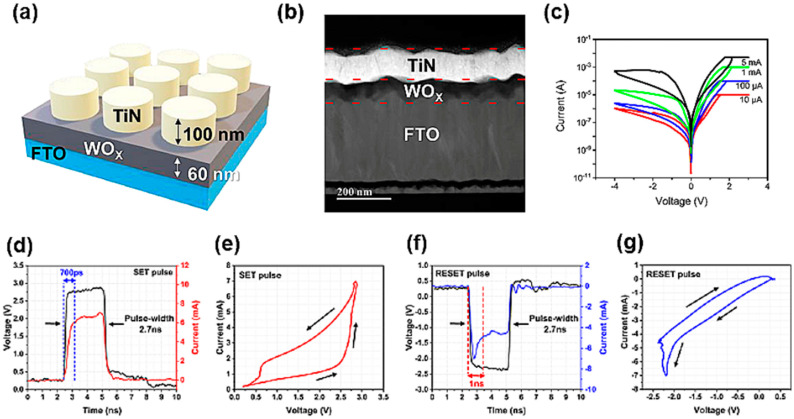
(**a**) Schematic of TiN/WO_X_/FTO device. (**b**) Cross-sectional TEM image. DC I–V curves of TiN/WO_X_/FTO RRAM device with different CC (compliance current) conditions. (**c**) I–V curves with varying CC (10 µA, 100 µA, 1 mA, and 5 mA) [4]. Copyright 2024, Elsevier. (**d**) Applied voltage pulse (black trace) and measured current (red trace) waveform for SET operation. The applied pulse has a pulse width of 2.7 ns, and the device switches in about 700 ps. (**e**) I–V plot of the data in (**d**) shows a change in resistance with the applied pulse. (**f**) Applied voltage pulse (black trace) and measured current (blue trace) waveform for RESET operation. (**g**) I–V plot of the data in (**f**) shows a change in resistance with the applied pulse [89]. Copyright 2024, nature.com.

RRAM is characterized by a MIM structure, which enables it to reversibly adjust its resistance state [77,78]. The structure of RRAM is shown in Figure 4a,b. RRAM devices operate based on resistive switching (RS), which involves transitions between a high-resistance state (HRS) and a low-resistance state (LRS) in response to external stimuli [79]. Additionally, RS enables multi-level cell (MLC) operations, which involve additional resistance states beyond the HRS and LRS [2,80]. RS operations can be classified based on various characteristics. For instance, digital RS involves an abrupt transition between the HRS and the LRS, whereas analog switching denotes a gradual change in resistance [81,82]. Figure 4c depict reversible transitions between the LRS and the HRS through analog switching, along with MLC operation. Furthermore, RS can be categorized into volatile and nonvolatile switching [83,84]. In volatile switching devices, RS is triggered by electrical stimuli, but the original state is promptly restored after the external stimulus is removed. In contrast, the resistance state of nonvolatile cells is maintained for a long period after the external stimulus is removed. Considering these characteristics, research on RRAM devices is being conducted from various perspectives [85,86]. Due to advantages such as high scalability (4F^2^), fast switching speed, low operating voltage, and a nonvolatile nature, RRAM devices are considered promising for next-generation applications involving nonvolatile data storage and artificial synapses [86]. Figure 4d–g demonstrate the exceedingly fast switching characteristics (within 1 ns) of RRAM. This fast switching is an important requirement for implementing artificial synapses, which requires fast processing with high power efficiency. Recently, the use of RRAM has also been proposed for other applications, such as memristors, logic gates, and switches [87,88].

### 3.1. RRAM Mechanism

The switching dynamics of memristor devices are closely related to their switching mechanisms. Various switching mechanisms have been demonstrated thus far, including the electrochemical metallization mechanism (ECM) [90], valence change mechanism (VCM) [91], and thermochemical mechanism (TCM) [92].

#### 3.1.1. ECM

**Figure 5:**
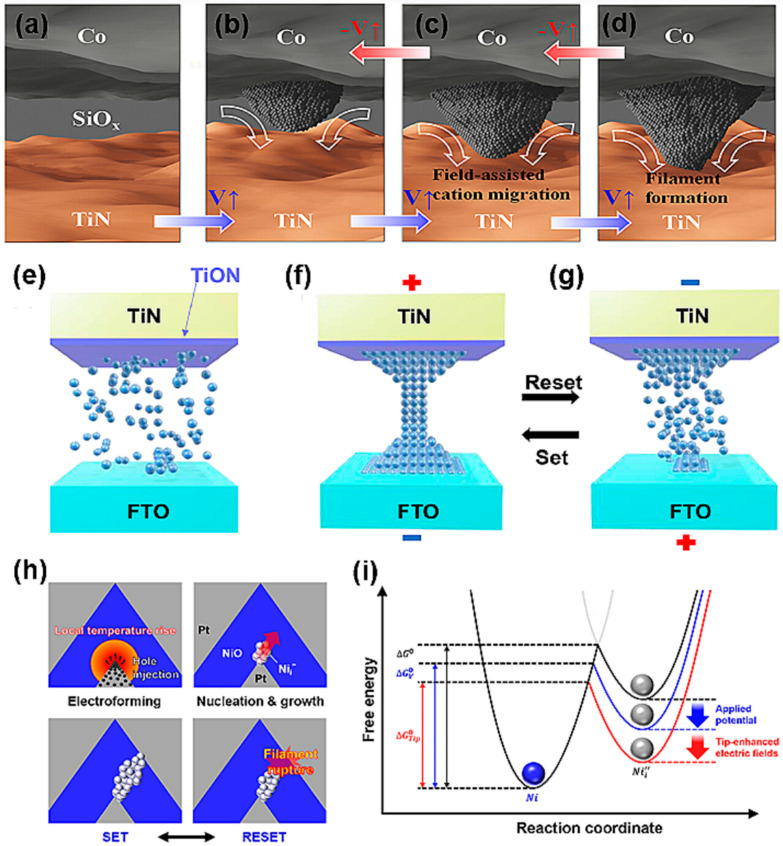
RS memory operation of a Co/SiO_X_/TiN cell. Filament morphologies based on observations of Co CBRAM in (**a**) pristine state, (**b**,**c**) HRS, and (**d**) LRS [95]. Copyright 2023, ACS Publications. Schematic of oxygen migration in TiN/WO_X_/FTO device: (**e**) initial state; (**f**,**g**) set and reset processes [4]. Copyright 2024, Elsevier. (**h**) Schematic illustration of RS behavior in pyramid-structured device. Nucleation and growth of the nickel (Ni) filament occur predominantly at the tip due to the locally accelerated Joule heating and facile hole injection, which result from the tip-enhanced electric field. (**i**) Energy diagram of reduction reactions at anode interface of nickel oxide (NiO) pyramid-structured RRAM [96]. Copyright 2021, Elsevier.

The concept of electrochemical metallization in ECM devices is based on the electrochemical redox reactions involving the active metal elements in a solid electrolyte within the device. Typically, the electrolyte is positioned between an electrochemically active electrode (such as Ag, Cu, or Ni) and an inert electrode (such as Pt, Pd, W, Au, or TiN) [93,94]. At the inert electrode, the cations are reduced to metal atoms, forming metallic conductive filaments (CFs) within the RS layer. Subsequently, the transition between the HRS and the LRS in the device is governed by the rupture/formation of these metallic CFs. The CFs can rupture partially or completely, depending on the strength of the applied electric field. For example, the use of cobalt (Co) as the active electrode and titanium nitride (TiN) as the passive electrode in electrochemical metallization (ECM) devices illustrates the mechanisms of resistance switching. As depicted in Figure 5a–d, when a sufficient positive voltage is applied, Co atoms migrate towards the TiN electrode through cation migration induced by the electric field. The process results in the crystallization of Co atoms, forming a conductive metallic bridge in contact with the TiN electrode, thereby placing the device in a low-resistance state (LRS). The formation and rupture of this conductive filament (CF) can be controlled by the applied electric field, which is crucial in determining the functional properties of ECM devices. Specifically, the device transitions to LRS with increased conductivity upon the application of a positive voltage due to CF formation. Conversely, applying a negative voltage can reset the device to a high-resistance state (HRS) by rupturing the CF. The reversible switching process, governed by the electric field, offers significant flexibility and efficiency in data processing and storage, impacting the performance and stability of ECM devices. Continued research and development in this area are expected to contribute to the advancement of next-generation memory technologies, particularly in applications requiring high speed and low power consumption.

#### 3.1.2. VCM

Oxygen vacancies in the active layer are essential for the switching of VCM devices. In these devices, the CF primarily consists of oxygen vacancies created through oxygen exchange reactions within the active layer. This exchange can occur throughout the active layer, where the movement of oxygen ions induces redox reactions by altering the valence state of cations, leading to stoichiometric changes in the oxides. Consequently, this anion migration-based mechanism is known as the VCM [6]. To achieve stable RS in VCM cells, an electroforming process is typically required [97]. During this process, highly dense oxygen vacancies are generated, and they migrate to form oxygen vacancy-rich filaments. Electrons then move along these filaments, which induces an LRS. When external stimuli are applied, these filaments can be ruptured, switching the device back to an HRS. The approximate mechanism of filament formation and rupture due to oxygen movement is illustrated in Figure 5e–g. This reversible switching process is fundamental to the operation of VCM-based devices.

#### 3.1.3. Thermochemical Mechanism

Figure 5h,i shows that switching mechanisms other than the ECM and VCM also exist. According to the dominant view, both electrical and thermal effects influence bipolar and unipolar RS processes [92]. In the RESET process, the abundant Joule heat generated in the CF provides energy, increasing the amplitude of atomic oscillation. This assists atoms in escaping from the CF, leading to its rupture. Devices governed by this mechanism can be considered unipolar [96]. In the TCM-type RESET process, Joule heating is widely believed to induce the rupture of the CF at its thinnest part. RS behavior can also stem from the charge trapping/detrapping effect, whereby carriers injected from the electrodes may be trapped in the active layer to form a space charge. This space charge induces RS by modulating the barriers via carrier injection from the electrodes or by altering the transport process of the charge carriers.

### 3.2. RRAM Materials

#### 3.2.1. Inorganic

**Figure 6:**
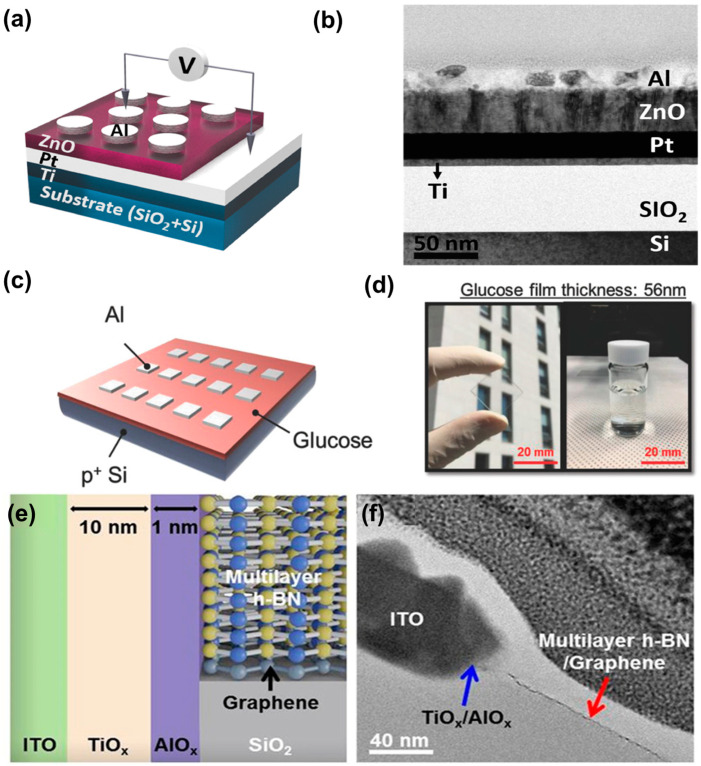
(**a**) Schematic of Al/ZnO/Pt device structure investigated in our research. (**b**) Cross-sectional HR-TEM images of Al/ZnO/Pt device [112]. Copyright 2022, Elsevier. (**c**) Device structure of glucose-based RRAM. (**d**) Photograph of glucose film with thickness information on glass substrate and synthesized solution [113]. Copyright 2018, Wiley Online Library. (**e**) Schematic showing cross-section of 2D material-based vertical RRAM. (**f**) Cross-sectional bright-field TEM image of as-prepared RRAM cell [114]. Copyright 2017, nature.com.

RRAM research has traditionally focused on the RS behavior observed in oxide-based semiconductor thin films, which has led to the development of various RRAM fabrication methods [79]. Binary oxides [98,99] and complex oxides [100,101] exhibit notable RS behavior. Due to their excellent performance, oxide-based RRAMs continue to be actively researched [88], as shown in Table 2. For example, RRAM can be fabricated using ZnO as an insulator, as shown in Figure 6a,b. Inorganic RRAMs have also been developed using nitrogen compounds and through doping with oxygen or nitrogen. The characteristics of these inorganic RRAMs are listed in Table 2. The resistance switching (RS) behavior observed in oxide-based semiconductor thin films has been the primary focus of research on inorganic RRAM. The exceptional performance of the materials continues to drive ongoing research, leading to the development of various fabrication techniques. However, a significant challenge in inorganic RRAM lies in balancing trade-offs among power consumption, switching speed, and retention time. Addressing the issues will require the exploration of novel materials and innovative strategies. For instance, research involving doping and the incorporation of nitrogen compounds holds potential for enhancing the performance of inorganic RRAM. Future studies should prioritize the integration of the materials and techniques to develop more advanced RRAM solutions.

**Table 2:** Summary of structural and performance parameters of inorganic RRAM devices.

Device Structure	V_SET_ (V)	V_RESET_ (V)	On/Off Ratio	Endurance	Retention	Synaptic Simulation	Reference
Ag/VO_X_/Pt	0.23	−0.07	10^3^	10^3^	12 h	-	[102]
Ti/ZrO_X_/HfO_X_/TiN	−2.0	2.4	10^3^	3 × 10^2^	10^4^	Potentiation /Depression (P/D)	[103]
TiN/HfO_X_/HfO_Y_/HfO_X_/TiN	0.27	−0.25	-	2 × 10^3^	10^4^		[104]
Ti/TaO_X_/indium tin oxide (ITO)	2.1	−2.1	10^2^	10^3^	10^4^	P/D, STDP, MNIST	[105]
Ag/TiO_X_N_Y_/Ga_2_O_3_/Pt	0.17	−0.057	10^5^	50	10^4^		[106]
Ti/NiFe2O4/Pt	0.75	−0.06	10^2^	10^2^		EPSC	[107]
Pt/HfO_X_/AlO_X_/TiN	2.2	−2.0	10^3^	10^4^	10^4^	STDP, SRDP, EPSC, PPF	[108]
Ta/SiN_X_/Pt	1.3	−1.1	10^3^	5 × 10^2^	3 × 10^2^		[109]
W/SiN_X_/n-Si	4.0	−4.0	10^3^		8 × 10^3^	MNIST (93.34%)	[110]
Ti/HfO_X_/Pt	0.5	−0.5	10^2^	10^4^	10^6^		[111]

#### 3.2.2. Organic

RRAM has primarily been developed using inorganic materials. However, the short lifespan of most electronic devices and memory components, especially those used in consumer applications, is often due to incorrect usage rather than component malfunction. The consequent disposal of these devices generates significant electronic waste, raising global concerns related to health, environmental impact, and material sustainability [115]. To address these issues, alternative engineering materials that are degradable, abundant, and environmentally friendly are being actively researched. A promising approach in this regard is the development of RRAM using organic materials [116]. Research in this area has focused on biological materials (such as silk protein and albumin) [117,118], polymer materials [119], and other organic substances. Similar to inorganic RRAM, bio-organic thin films are sandwiched between two metal electrodes (top and bottom electrodes). For example, RRAM can be manufactured using glucose as an insulator, as shown in Figure 6c,d. Studies have shown that RRAM devices fabricated using inorganic materials tend to exhibit more stable switching behavior, lower energy consumption, and longer retention times compared with those created from organic materials. Moreover, as the pursuit of organic RRAM is driven by its potential for greater sustainability and reduced environmental impact, it represents a key area of ongoing research. Additionally, Table 3 shows that organic RRAMs sometimes outperform their inorganic counterparts. The development of organic RRAM represents a key approach to addressing environmental concerns related to electronic waste. By utilizing biodegradable materials, researchers aim to enhance the sustainability and lifespan of electronic devices. However, compared to inorganic counterparts, organic RRAM typically exhibits inferior switching stability, higher energy consumption, and shorter retention times. Such limitations are often attributed to the inherent physical properties and chemical stability of organic materials. Future research must focus on overcoming the challenges and improving the performance of organic RRAM through the development of novel materials and device architectures. The sustainability of organic RRAM could play a pivotal role in its commercial success.

#### 3.2.3. Low-Dimensional Materials

While oxide-based RRAM devices demonstrate different desirable properties, realizing a functional memory material that possesses all the desired attributes remains a significant challenge. For example, the most critical trade-offs, such as power–speed, speed–retention, and endurance–retention, cannot be eliminated by any oxide-based RRAM device. The electrical properties of these devices can be influenced by the chemisorption of O2 molecules, which reduces electrical conductivity. Additionally, existing functional materials for RRAM are limited by scaling bottlenecks. Therefore, new functional materials must be researched to facilitate the development of commercially viable next-generation flexible and wearable RRAM devices. Recent advances have led to extensive research on the use of low-dimensional materials, including 2D, 1D, and 0D materials, for RRAM devices. Specifically, 2D materials, known for their excellent electrical performance and unique characteristics, such as flexibility, transparency, and thermal stability, have been widely studied. Examples include graphene [130], TiO_2_ [131], h-BN [132], and certain organic materials [133]. Graphene, in particular, has been the most extensively researched material and is one of the first 2D materials utilized for RS devices. Figure 6e,f presents examples of graphene-based RRAM. In addition to 2D materials, 0D materials, such as graphene oxide QDs [134], and 1D materials, such as carbon nanotubes [135] and metal oxide nanowires (NWs) [136], have been proposed for RS devices. In applications such as photovoltaic memory devices, LEDs, and bioimaging, 0D QDs are crucial due to their unique electronic and optical properties [137]. The performance of RRAM based on these low-dimensional materials has been promising, and research on these materials continues to be actively conducted to overcome the limitations of oxide-based RRAM devices. Table 4 summarizes the characteristics of RRAM based on low-dimensional materials. The future of RRAM technology is likely to be driven by advancements in low-dimensional materials. The unique optical and electrical properties of 0D, 1D, and 2D materials offer significant potential for next-generation memory devices. Graphene, characterized by outstanding electrical performance, flexibility, and transparency, has emerged as a critical material in the development of RRAM. However, several technical challenges must be addressed before low-dimensional materials can be commercialized. Researchers should prioritize leveraging the advantages of the materials to develop novel memory devices and overcome the limitations of conventional oxide-based RRAM. Future research should focus on optimizing the capabilities of low-dimensional materials to create commercially viable RRAM solutions.

## 4. QD-Based RRAM

Among the various materials used in RRAM, QDs are being actively researched. RRAM incorporating QDs offers several advantages, including extremely fast operation [148], multilevel data storage capabilities [149], and very low operating voltages [134]. Moreover, RS characteristics can be realized in nanoscale QDs, which can enable the implementation of ultrahigh-density nano memory [10,150]. In particular, QD-based RRAM can exhibit optical tunability [16,151], which showcases the potential of this technology.

**Figure 7:**
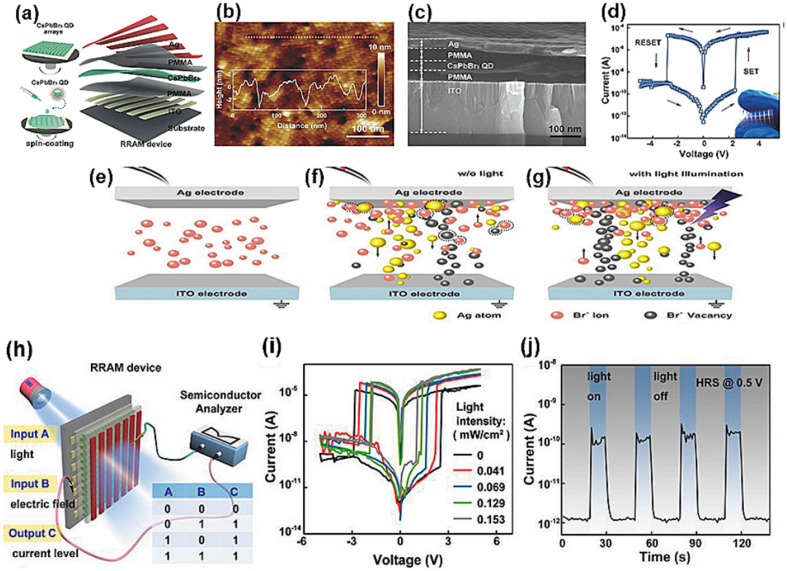
(**a**) Schematic illustration of CsPbBr3 QD-based RRAM device fabrication via all-solution process. (**b**) Atomic force microscope image showing topography of CsPbBr3 QDs’ arrays with height profile of surface. (**c**) Cross-sectional scanning electron microscope image showing side view of Ag/PMMA/CsPbBr3 QDs/PMMA/ITO device. (**d**) Typical I–V plot of the device; inset shows photograph of the as-prepared device. The photoresponse curve of the RRAM device in the HRS was measured at 0.5 V with UV light pulse stimulation. (**e**) Illustration of RS in the initial state, (**f**) during the SET process under dark conditions, and (**g**) during the SET process under UV illumination. (**h**) Schematic illustration of CsPbBr3 QD-based logic OR device. (**i**) Typical I–V characteristics of ITO/PMMA/CsPbBr3/PMMA/Ag-structured device measured in the dark or under a UV lamp. (**j**) Photoresponse curve of the RRAM device in HRS, measured at 0.5 V with UV light pulse stimulation [152]. Copyright 2018, Wiley Online Library.

As shown in Figure 7a–c, QD-based RRAM comprises QDs inserted into the insulator layer of a traditional MIM structure. The resistance state of the QD-based film can be switched via an external bias stimulus; the reversible transitions between the LRS and the HRS in a typical QD-based RRAM are depicted in Figure 7d. As shown in Figure 7e,f, QDs generally act as charge traps, functioning primarily based on mechanisms such as ion migration and charge trapping. Ion migration produces localized CFs in the switching layer. Depending on the type of ion, ion migration can be classified into active metal ion migration and oxygen vacancy migration. Under an external electric field, metal ions from the active electrode or oxygen vacancies within the QD-based film are thermally and/or electrically activated. The accumulation of these driven metal ions or oxygen vacancies forms a conductive pathway within the QD-based device, switching the device to a LRS. Conversely, applying a reverse electric field can rupture the conductive pathway, switching the device back to a HRS. Charge trapping is another common operation mechanism for QD-based RRAM [14]. Under strong electric fields, electrons or holes are injected via tunneling and are primarily captured by the trapping centers in the QDs, altering the electrostatic barrier characteristics of the memory device.

QDs are also used to enhance the characteristics of RRAM. For example, inserting a QD film into the insulator layer can provide electron traps, leading to performance improvements [15]. Alternatively, QDs can be combined with highly insulating materials such as polymethyl methacrylate (PMMA), where they can serve as a means of electron transport [153,154]. Additionally, graphene QDs (GQDs), which combine the 2D structure of graphene with quantum confinement effects, are utilized in RRAM applications. Due to the narrow bandgap of QDs, they can also be used in photonic RRAM. The photoelectric effect induced by UV light can lower the operating voltage required by a device [16]. Moreover, Figure 7g shows that the induced photoelectrons from QDs help form filaments. Figure 7h–j shows that QD RRAM can be used as an OR gate under light stimulation and that the device can be controlled with light pulses. In addition, the I–V curve varies with the light intensity. This highlights the possibility of developing optically tunable artificial synapses. Table 5 summarizes the performance data of existing QD-based RRAM devices.

## 5. Application

### 5.1. Artificial Synapses

Human neural networks consist of neurons (nerve cells) and synapses (junctions where signals are transmitted between neurons). The transmission, processing, and storage of information rely on the mechanism of synaptic plasticity. Research is being conducted to develop neuromorphic systems that can mimic such human neural networks while performing at low power, high speed, and high efficiency, to overcome the limitations of the traditional von Neumann architecture. The use of QD-based RRAM devices as artificial synapses has been extensively studied [24,165]. Due to advantages such as optical tunability, low cost, fast operation, low operating voltage, and long retention, QD-based RRAM is a promising candidate for implementing artificial synapses [170].

**Figure 8:**
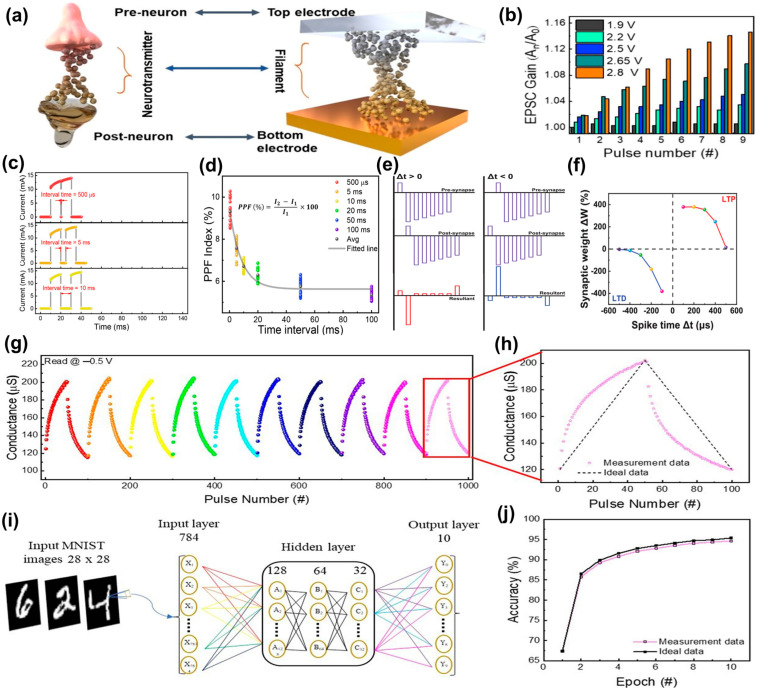
(**a**) Schematic showing imitation of a synaptic neural structure through device synaptic plasticity. (**b**) EPSC gain values in response to different programming pulse amplitudes [174]. Copyright 2024, ACS Publications. (**c**,**d**) PPF characteristics of TiN/ZnO/NiO/Pt device: statistical distribution of PPF as a function of interval time [175]. Copyright 2023, Elsevier. (**e**) Pulse schematic for STDP. (**f**) Result of planar STDP measurement [176]. Copyright 2024, Wiley Online Library. (**g**,**h**) Cycle-to-cycle multiple potentiation and depression curves; (**i**) deep neural network simulation framework for MNIST pattern recognition; (**j**) pattern recognition accuracy of synaptic device over 10 consecutive epochs [175]. Copyright 2023, Elsevier.

Figure 8 shows the results of various simulations of mimicking the human brain. For example, excitatory postsynaptic current (EPSC) and inhibitory postsynaptic current (IPSC) must be simulated through RRAM. The resulting electrical signal generated by the postsynaptic neuron is called an EPSC or IPSC. The EPSC/IPSC values are proportional to the weight changes, with higher EPSC/IPSC values implying stronger weight changes in synaptic plasticity [171]. Additionally, PPF and paired-pulse depression (PPD) must be simulated to mimic short-term plasticity behavior [172], as well as STDP and spike-rate-dependent plasticity (SRDP), which are typical learning rules [173]. In accordance with guidelines stipulated by the Modified National Institute of Standards and Technology (MNIST), accuracy can be determined based on linear potentiation/depression values. Upon closer examination of each figure, the behavior of memristor devices, as shown in Figure 8a, can be likened to the learning process in synaptic neurons, where resistance changes due to filament stability between memristor electrodes resemble the learning dynamics in synaptic networks influenced by neurotransmitter intensity. The correlation validates the potential for synaptic devices that leverage the characteristics of long-term memory (LTM) and short-term memory (STM). Learning and memory functions in neural systems are governed by a linear change in synaptic weight, as evidenced by the excitatory postsynaptic current (EPSC) gain values for different programming pulse amplitudes within the 1.9–2.8 V range, depicted in Figure 8b. The trend shows that increasing programming pulse amplitude with a 1 μs pulse width leads to an increase in EPSC gain, reflecting variations in device conductivity. The finding highlights the presence of spike amplitude-dependent plasticity, where the memristor’s weight can be modulated in response to pulse inputs, a key feature in neuromorphic computing. The short-term potentiation (STP) properties of artificial synapses can be demonstrated through paired-pulse facilitation (PPF) measurements, particularly in the TiN/ZnO/NiO/Pt device. The PPF phenomenon, as illustrated in Figure 8c, indicates that modifying the time intervals between successive pulse stimuli can adjust synaptic weights. The confirmation is obtained by observing current variations after applying two identical pulses with varied time intervals (amplitude: −4.5 V, width: 10 ms). The PPF index, calculated based on the rate of change between the first and second pulses over 20 cycles, is shown in Figure 8d. The PPF index, representing the ratio of the difference between the average pre-pulse current (I_1_) and average post-pulse current (I_2_), reveals how the synaptic weight adjustment fluctuates with time intervals, changing exponentially as the interval increases. The results suggest that the artificial synapse effectively replicates the PPF function as STM, with a noticeable increase in the second pulse’s rate of change relative to the first when shorter intervals are applied. However, the current rate of change tends to decrease with longer stimulus intervals, indicating unstable memory formation. The findings provide fundamental data for designing and optimizing artificial synapses, which could contribute to the development of AI systems based on neural networks. Spike-timing-dependent plasticity (STDP) is a key physiological mechanism that describes how synaptic weights change depending on the timing of spikes between synapses. The change in synaptic weight (ΔW) in this process is determined by the time difference (Δt) between the spike timings of the presynaptic and postsynaptic neurons. As illustrated in Figure 8e, when the presynaptic neuron fires before the postsynaptic neuron (Δt > 0), a negative pulse is applied, leading to long-term potentiation (LTP) and an increase in synaptic weight. Conversely, when the postsynaptic neuron fires before the presynaptic neuron (Δt < 0), a positive pulse is administered, causing long-term depression (LTD) and a decrease in synaptic weight. The outcomes of spike applications at various timings, as shown in Figure 8b, demonstrate how the STDP rule gradually alters synaptic weights, reflecting the complex dynamics of neural systems. In human physiology, nociceptors beneath the skin transmit injury-related information to the brain, influencing synaptic connections and potentially leading to the formation of strong synaptic bonds when neurons are injured. QDs are expected to play a significant role in the advancement of artificial synapses. Photonic memories based on inorganic QDs, such as CsPbBr_3_, offer excellent optical characteristics and unique connectivity, which may alleviate the von Neumann bottleneck in unconventional computing. The unique optical properties of QDs are particularly advantageous for simulating biological synapses, allowing for the replication of key neural network mechanisms such as spike-timing-dependent plasticity and both long- and short-term plasticity. By leveraging the wavelength-responsiveness of QDs, synaptic weights can be precisely tuned, enhancing the complexity of information processing. Furthermore, charge capture and release processes in QD-based photonic memory enable effective optical programming and electrical erasing, allowing artificial synapses to function flexibly across various contexts and process information in a manner akin to biological brain networks. The dynamic reconfiguration of synapses, enabled by the distinctive properties of QDs, can significantly improve the efficiency of information processing and storage, making them a crucial component in the development of advanced neuromorphic computing systems. Overall, QD technology has the potential to greatly enhance the functionality of artificial synapses and play a pivotal role in the future of artificial intelligence and other fields by emulating the sophisticated information processing capabilities observed in neural networks. Quantum dot technology is believed to contribute to the advancement of artificial intelligence by enabling more efficient information processing techniques and fostering the development of more effective systems.

### 5.2. Other Applications

Considering its low power consumption and small size, RRAM can be used for data storage and processing in embedded systems, such as IoT devices, sensors, and wearable devices [177]. QD-based RRAM can be more effective in this regard because of its lower power consumption relative to traditional RRAM. It can also be employed as an OR gate under light stimulation [165]. In addition, QD-based RRAM is expected to be widely applicable because of advantages such as low-voltage operation, fast switching speed, optical tunability, and small device size.

## 6. Conclusions

QDs are notable for their unique optoelectronic properties, solution processability, and suitability for RRAM fabrication. QD-based RRAM is gaining attention for its fast switching speed, low operating voltage, and optical tunability; therefore, it represents a promising solution for artificial synapse implementation. The paper described the material properties and synthesis methods for QDs. Due to their nanoscale size, QDs exhibit quantum confinement effects and emit light of specific wavelengths when stimulated externally. Synthesis methods for QDs include colloidal synthesis and sol–gel synthesis. However, recent efforts have focused on developing more environmentally friendly and cost-effective techniques. Understanding the size and surface ligands of QDs is crucial for comprehending their properties. Modulating factors such as the ligand binding strength and molecular structure can enhance the performance of QD-based devices, and the emission wavelength of QDs can be controlled by adjusting their size.

The importance of RRAM and its development direction were emphasized, and its operation mechanisms were described as well. RRAM is considered a next-generation device for nonvolatile data storage and artificial synapse application due to its high scalability and fast switching. The operation mechanisms of RRAM include the ECM and VCM, and understanding these mechanisms is essential for leveraging the benefits of RRAM. Various materials are used to fabricate RRAM, including organic, inorganic, and low-dimensional materials. Specifically, QDs have been a recent focus due to their advantages, which include optically tunable properties and the potential for ultra high-density nano memory implementation. QDs act as traps that provide electron migration paths, enhancing RRAM performance.

Finally, this paper described the potential applicability of QD-based RRAM as artificial synapses. QD-based RRAM can simulate EPSC, PPF, and STDP, which demonstrates its potential as a material for artificial synapse implementation.

## Figures and Tables

**Table 1:** Synthesis methods and properties of QDs.

Synthesis Method	Type of QD	Size of QD (nm)	Bandgap (eV)	Properties	Reference
Colloidal	CdSe/CdS	1.2	-	Removal of organic ligands and formation of bridges with CdS/ZnS	[54]
Chemical precipitation	ZnO	50	-	Eu^3+^ and Er^3+^ ions are doped into the ZnO lattice to reduce grain size	[55]
Sol–gel	CdSe	-	>1.74	No surface ligands and suppressed particle-to-particle interactions	[56]
Hydrothermal	ZnS	4.3	-	ZnS nanoparticles uniformly distributed on the graphene surface	[57]
Colloidal	ZnS	4.7 ± 0.1	3.74 ± 0.02	Encapsulation with chitosan provides colloidal stability	[58]
Colloidal	ZnS	10	-	ZnS nanoparticles uniformly distributed on the graphene surface, bandgap effect due to high defect density	[59]
Colloidal	CdSe	4.2	1.9	High luminous efficiency, prolonged stability	[60]
Thermal decomposition	CdS	2.44	-	Excellent bonding power with mesoporous TiO_2_ film	[61]
Thermal decomposition	PbSe	5	1.3	Presence of organometallic ligands, strong luminescence	[62]
Microemulsion	Silicon nanocrystals	1.4–10	-	Hydrophilic and hydrophobic surfaces can be modified	[63]
Hydrothermal	G–ZnS	10	3.7	High surface defect concentration	[64]
Self-Assembly	InAs/GaAs	-	-	QD molecules	[65]
Sol–gel	ZnO	5	-	High thermal and temporal stability, voltage adjustable in combination with UV chip (PLQY 63.7%)	[66]
Sol–gel	PbS	2.9–3.1	1.2	Strong near-infrared photoluminescence, ultrafast electronic relaxation properties, and cubic-phase PbS crystallization	[67]
Sol–gel	Zn_1−x_Mg_x_O	3.8–4.7	3.74–3.92	Oxygen vacancies and organic residues on the surface	[68]

**Table 3:** Summary of structural and performance parameters of organic RRAM devices.

Device Structure	V_SET_ (V)	V_RESET_ (V)	On/Off Ratio	Endurance	Retention	Synaptic Simulation	Reference
FTO/ZnO/CH_3_NH_3_Pb_1-X_Bi_X_Br_3_/Pt	1.05	−0.82	10^5^	10^2^	10^4^		[120]
Al/MAPbl_3_/Al	1.66	−0.47	10^6^	5 × 10^2^	10^4^		[121]
ITO/P_3_HT:PCBM/Al	0.5	−1.2	4 × 10^2^	47			[122]
ITO/trimesic acid and poly(4-vinylpyridine) (PVP)/Al	2.5	−2.5	7.5 × 10^4^	5 × 10^2^	10^5^	PPF	[123]
Al/PCBM + PVP/Al	6	−6	10^3^		10^5^		[124]
Ag/chitosan/FTO	5	−5	10^2^	2 × 10^2^	10^4^		[125]
Ag/Gelatin/HfO_2_/ITO	2.2	−2.0	10^5^	10^2^	10^4^		[126]
Ag/Ag-TCNC/FTO	0.2	−0.2	10^4^	10^4^	10^4^	LTP/LTD, SRDP, EPSC, PPF/PPD	[127]
Al/P3HT:4CzlPN,2CzPN/ITO	8	−8	10^5^	1.5 × 10^2^	5 × 10^4^		[128]
Ag/ZnO/P3HT-PCBM/ITO	2	−0.9	10^5^	5 × 10^2^	10^4^		[129]

**Table 4:** Summary of structural and performance parameters of RRAM devices based on low-dimensional materials. (The term low-dimensional material is highlighted in bold to emphasize its importance).

Device Structure	V_SET_ (V)	V_RESET_ (V)	On/Off Ratio	Endurance	Retention	Synaptic Simulation	Reference
ITO/Al_2_O_3_/**PdSe_2_**/Al_2_O_3_/TaN	1.2	−1.0	10^2^	8 × 10^2^	10^4^	EPSC/IPSC, PPF/PPD, STDP	[138]
Au/**Bi_2_O_2_Se**/**Bi_2_SeO_X_**/Au	1.6	−0.8	10	10	5 × 10^3^	EPSC, PPF/PPD	[139]
Au/**ReSe_2_**/Au	4	−2	10^4^	2 × 10^2^	10^4^	MNIST (95.71%)	[140]
Pt/**ZTO**/Ti/Au	3	−1.1	10^3^	10^2^	10^5^		[141]
Au/Ti/**2D SnO_2_**/SiO_2_/p^+^-Si	15	−15	10	10^3^	10^4^	P/D, EPSC, MNIST (92.25%)	[142]
Al/CsPbBr_3_ QDs/**MoS_2_**/FTO	1.0	−1.0	12	10^2^	-		[143]
ITO/CdSe:ZnS QDs/**MoS_2_**/TiO_2_/Pt	2	−2	10	10^2^		P/D	[144]
Al/**GO**/ITO	3.5	−2.0	20	50	10^4^	P/D	[145]
ITO/**MAPbI_3−X_Cl_X_ 2D perovskite**/Al	0.79	−0.77	10^3^	3 × 10^2^	10^4^		[146]
Ti/TiO2/**Mo-ReS_2_**/Ti	3	−3	10		10^3^	P/D, MNIST (91%)	[147]

**Table 5:** Summary of structural and performance parameters of QD-based RRAM devices.

Device Structure	V_SET_ (V)	V_RESET_ (V)	On/Off Ratio	Endurance	Retention	Synaptic Simulation	Reference
Al/PMMA/ZnO QDs/PMMA/ZnO QDs/PMMA/FTO	1.1	−1.5	70	2 × 10^2^	5 × 10^3^		[155]
ITO/InP:ZnSe:ZnS QDs/PMMA/Al	1.06	−2	35.5	50	10^4^	P/D, MNIST (91.46%)	[19]
Al/PMMA/WS_2_ QDs/PMMA/FTO	4	−8	10^3^	2 × 10^2^	10^4^		[156]
Al/CdSe QDs-PVP/Al	0.61	−0.5	6.1 × 10^4^	1.5 × 10^2^	35 × 10^3^		[28]
ITO/CdS QDs-PVP/Al	1.08	−0.72	4.7 × 10^4^	3 × 10^2^	6 × 10^4^		[157]
W/MoS_2_ QD/ZnO/Ag	0.12	−0.25	10^2^	2 × 10^2^	10^4^		[158]
Ag/Ta_2_O_5_/MoS_2_ QDs/Pt	0.3	−0.14	10^7^	5 × 10^2^	10^4^		[159]
Al/MoS_2_ QDs-PVP/ITO	1.45	−1.35	10^5^	2.5 × 10^2^	27 × 10^3^		[160]
ITO/PEDOT/CdSe:ZnS QDs/ZnO/Al/Al_2_O_3_/ CdSe:ZnS QDs/Al	2.4	−4.6	2.5 × 10^3^	2 × 10^2^	36 × 10^4^		[161]
Ag/PMMA:CQDs/FTO	1.1	−1.5	1.5 × 10^2^	2 × 10^2^	10^4^		[162]
Au/PMMA/PMMA:MoS_2_ QDs/PMMA/FTO	0.5	−0.9	10^2^	2 × 10^2^	10^4^		[163]
ITO/CdSe (QDs)/PMMA/Al	1.6	−0.5	10^5^	10^3^	10^4^		[16]
Ag/Ga_2_O_3_/NQDs/Pt	0.19	−0.12	10^6^	10^9^	3.5 × 10^6^	STDP, PPF,	[24]
Ag/PMMA/CsPbBr_3_ QDs/PMMA/ITO	1.1	−1.7	10^5^	5 × 10^3^	4 × 10^5^		[164]
Ag/InZnS QDs/TiO_2_/Pt	0.08	−0.05	10^5^	2 × 10^2^	10^4^	P/D, PPF	[153]
Ag/TiO_2_ NWs:ZnO QDs/FTO	3	−3		15		EPSC, PPF/PPD, P/D, MNIST (88.9%)	[165]
Ag/C_15_ZO QDs/Pt	0.41	−0.23	10^5^	10^4^	10^4^	P/D, PPF, MNIST (92.6%)	[166]
FTO/MoS_2_ QDs/Al	2	−2	10^4^	60	10^3^	P/D, PPF/PPD	[167]
Al/PMMA:(InP/ZnSe/ZnS QDs)/ITO/PEN	2.1	−3.1	8.5 × 10^3^	10^2^	10^4^		[168]
Al/PMMA/BP QD/PMMA/Al	2.9	−2.6	3 × 10^7^	10^2^	10^4^		[169]
